# Habitat-specific shaping of proliferation and neuronal differentiation in adult hippocampal neurogenesis of wild rodents

**DOI:** 10.3389/fnins.2013.00059

**Published:** 2013-04-18

**Authors:** Nicole Cavegn, R. Maarten van Dijk, Dominik Menges, Helene Brettschneider, Mashudu Phalanndwa, Christian T. Chimimba, Karin Isler, Hans-Peter Lipp, Lutz Slomianka, Irmgard Amrein

**Affiliations:** ^1^Institute of Anatomy, University of ZurichZurich, Switzerland; ^2^Department of Zoology and Entomology, University of PretoriaHatfield, South Africa; ^3^National Zoological Gardens of PretoriaPretoria, South Africa; ^4^Western Cape Nature Conservation Board (CapeNature)Cape Town, South Africa; ^5^DST-NRF Centre of Excellence for Invasion Biology, University of PretoriaHatfield, South Africa; ^6^Anthropological Institute and Museum, University of ZurichZurich, Switzerland

**Keywords:** Muridae, neurogenesis, hippocampus, CA4, gender, habitat, Ki67, doublecortin

## Abstract

Daily life of wild mammals is characterized by a multitude of attractive and aversive stimuli. The hippocampus processes complex polymodal information associated with such stimuli and mediates adequate behavioral responses. How newly generated hippocampal neurons in wild animals contribute to hippocampal function is still a subject of debate. Here, we test the relationship between adult hippocampal neurogenesis (AHN) and habitat types. To this end, we compare wild Muridae species of southern Africa [Namaqua rock mouse (*Micaelamys namaquensis*), red veld rat (*Aethomys chrysophilus*), highveld gerbil (*Tatera brantsii*), and spiny mouse (*Acomys spinosissimus*)] with data from wild European Muridae [long-tailed wood mice (*Apodemus sylvaticus*), pygmy field mice (*Apodemus microps*), yellow-necked wood mice (*Apodemus flavicollis*), and house mice (*Mus musculus domesticus*)] from previous studies. The pattern of neurogenesis, expressed in normalized numbers of Ki67- and Doublecortin(DCX)-positive cells to total granule cells (GCs), is similar for the species from a southern African habitat. However, we found low proliferation, but high neuronal differentiation in rodents from the southern African habitat compared to rodents from the European environment. Within the African rodents, we observe additional regulatory and morphological traits in the hippocampus. Namaqua rock mice with previous pregnancies showed lower AHN compared to males and nulliparous females. The phylogenetically closely related species (Namaqua rock mouse and red veld rat) show a CA4, which is not usually observed in murine rodents. The specific features of the southern environment that may be associated with the high number of young neurons in African rodents still remain to be elucidated. This study provides the first evidence that a habitat can shape adult neurogenesis in rodents across phylogenetic groups.

## Introduction

Adult hippocampal neurogenesis (AHN) in wild living mammals shows large species-specific variation. It can either be absent as in bats (Amrein et al., 2007), or exceptionally high as in red foxes (Amrein and Slomianka, 2010). All wild rodents that have been studied show AHN to various degrees [for a review see Amrein et al. (2011)], but no common behavioral, ecological, or taxonomic feature has been identified that can explain the variation between species. Spatial orientation requirements have been suggested to depend on AHN, as territory size in wild rodents correlates with proliferation (Amrein et al., 2004b). AHN may however differentially impact on performance in specific spatial tasks (Saxe et al., 2007) and only weak or no correlations have been found between AHN and demands on spatial memory within and across species of food hoarding rodents (Barker et al., 2005; Johnson et al., 2010). Recently, the idea that behavioral flexibility may depend on and express itself in the form of AHN (Garthe et al., 2009; Amrein et al., 2011) has been interpreted as a means for the ontogenetic and phylogenetic adaption to changing habitats (Kempermann, 2012).

To test the relationship between AHN and habitat types, we investigated AHN in wild small rodents from different genera and divergent habitats by comparing animals from a generally warm southern African habitat with those from a generally cold European environment. The wild southern African rodents (Muridae) included the Namaqua rock mouse (*Micaelamys namaquensis*) and the phylogenetically closely related Red veld rat (*Aethomys chrysophilus*), representing the subfamily Murinae (Old world rats and mice), the highveld gerbil (*Tatera brantsii*) representing the subfamily Gerbillinae (gerbils, jirds, and relatives), and the southern Africa spiny mouse (*Acomys spinosissimus*) representing the subfamily Deomyinae (spiny mice, forest mice, and relatives) (Skinner and Chimimba, 2005). For European rodents we used data from wild rodents of the family Muridae (subfamily Murinae) that included long-tailed wood mice (*Apodemus sylvaticus*), pygmy field mice (*Apodemus microps*), yellow-necked wood mice (*Apodemus flavicollis*), and house mice (*Mus musculus domesticus*) from previous studies in our laboratory.

All southern African rodents were sampled from the same locality, which is a mostly rocky and sandy habitat with strong seasonal temperature variation between 0°C in winter and up to 45°C in summer. Tissues of all southern African samples were immunohistochemically stained against proteins specifically expressed by either proliferating cells [Ki67; Starborg et al. (1996)] or differentiating cells of neuronal lineage [Doublecortin, DCX; Matsuo et al. (1998)]. The total number of granule cells (GCs) and the number of pyknotic cells were determined in Giemsa stained material. Inter-specific comparisons were conducted based on estimates of the total numbers of proliferating cells and neuronally differentiating cells normalized to the total GC numbers. For habitat classification, we applied the Köpper–Geiger climate classification which is based on monthly precipitation and temperature (Peel et al., 2007). The South-African habitat at the sampling site is described as temperate, with dry winter and hot summer (Cwa). The European habitats (Eastern Russia and Europe) are classified equally as cold, with warm summers and no dry seasons (Dfb).

## Materials and methods

### Animals

Namaqua rock mice, red veld rats, gerbils and spiny mice (*n* = 33) (for details see Table 1) were live-trapped in early spring at Goro Game Reserve [Limpopo Province, South Africa; (22° 58′S, 22° 57′S; 29° 25′E, 29° 24′E)] using Sherman live traps (H.B. Sherman Traps Inc., FL, USA). Animal trapping and handling followed the guidelines of the American Society of Mammalogists (Gannon and Sikes, 2007) and was approved by the Animal Ethics Committee of the University of Pretoria, South Africa (Ethics Clearance Number EC028-07) and the CITES and Permits Management Office, Department of Environmental Affairs, Limpopo Province, South Africa (Permit number CPM-333-00002). Animals were identified morphologically (see below for a more detailed genetic identification procedure), sexed, and perfused immediately after trapping.

**Table 1:** **Morphological data and cell number estimates (calculated for one hemisphere) of the four southern African rodent species Namaqua rock mouse, red veld rat, representing the subfamily Murinae (Old world rats and mice), the highveld gerbil, representing the subfamily Gerbillinae (gerbils, jirds and relatives), and the southern African spiny mouse, representing the subfamily Deomyinae (spiny mice, forest mice, and relatives)**.

		**Brain weight [g]**		**Body weight [g]**		**Lens weight [mg]**		**Total granule cells**		**Ki67**		**DCX**		**Apoptotic cells**	
**Species**	**Sex(N)**	**Mean**	***SD***	**Mean**	***SD***	**Mean**	***SD***	**Mean**	***SD***	**Mean**	***SD***	**Mean**	***SD***	**Mean**	***SD***
Namaqua rock mouse	F(11)	0.88	0.07	38.2	8.38	34.7	5.06	1,131,984	228,780	2264	473	24,682	7248	80	70
	M(11)	0.93	0.06	38.7	3.70	33.2	2.04	1,118,548	240,856	2763	311	32,836	5731	82	64
Red veld rat	F(4)	0.91	0.07	39.1	8.32	35.8	4.78	1,044,985	294,795	1809	355	21,600	6855	99	96
	M(2)	0.90	0.18	41.0	5.66	33.3	1.62	1,076,667	8228	2937	352	32,025	9864	36	8
Highveld gerbil	F(2)	1.15	0.01	62.0	0.64	58.8	2.67	1,261,294	108,927	2400	458	25,425	1591	84	17
	M(1)	1.27	–	59.0	–	41.1	–	1,344,295	–	2874	–	41,350	–	66	–
Spiny mouse	F(1)	0.65	–	19.3	–	12.5	–	2,069,250	–	9940	–	51,000	–	NA	NA
	M(1)	0.63	–	23.3	–	13.0	–	2,964,834	–	11,155	–	34,250	–	NA	NA

After deep anesthesia using Pentobarbital (50 mg/kg body weight), animals were perfused transcardially with heparinized phosphate buffered saline (PBS) followed by sodium sulfide solution and 4% paraformaldehyde (PFA) with 15% of a saturated solution of picric acid. Brains, femurs, and eye lenses were collected and post-fixed in 4% PFA overnight. Right brain hemispheres were transferred into 30% sucrose in PBS for immunohistochemistry. The left hemispheres were kept in fresh PFA until 2-hydroxyethylmethacrylat (HEM) embedding. Femurs and eye lenses were transferred into PBS/azide (500 mg/l) until further processing. Reproductive status of females was determined by inspecting uteri for pregnancy marks, and females where classified as nulliparous (no former pregnancy) or parous for females that had at least one litter before. None of the females were pregnant or lactating at the time of trapping.

### Histology

**Figure 1:**
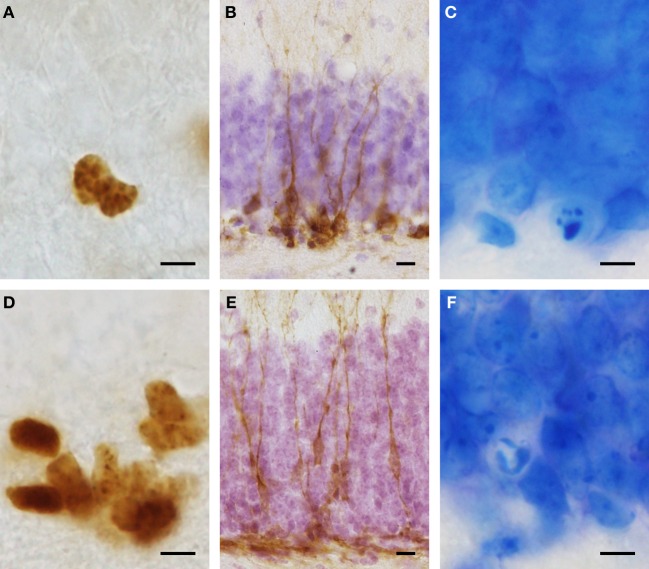
**Representative images of immunohistological stainings for proliferating cells (Ki67: A,D) and young cells of the neuronal lineage (DCX: B,E) in Namaqua rock mouse (A–C) and spiny mouse (D–F).** Condensed chromatin typical for dying cells **(C,F)** can be easily differentiated in Giemsa stained sections and were used for cell death quantification. Note that granule cells in the spiny mouse **(E,F)** are considerably smaller than those in the Namaqua rock mouse **(B,C)**. Scale bar **(A,C,D,F)**: 5 μm; **(B,E)**: 10 μm.

For immunohistochemistry, 40 μm sagittal serial sections of frozen right hemispheres were collected and kept at −20°C in a cryo-protection solution till further processing. Free-floating sections were stained for DCX, Ki67 (Figure 1) and calbindin. Between all steps, sections were washed with Tris-Triton [TBS (pH = 7.4)/0.05% Triton] and, after incubation with primary antibody, with TBS only. For epitope retrieval, sections were transferred to citrate buffer (Dako) and either shortly microwaved (DCX and Calbindin) or heat-treated (40 min at 94°C for Ki67). To inhibit endogenous peroxidase activity, sections were incubated in 0.06% H_2_O_2_ for 15 min. After pre-incubation in Tris-Triton, 2% normal serum, 0.2% Triton, and 0.1% bovine serum albumin (BSA), sections were incubated in DCX (polyclonal goat IgG sc-8066, Santa Cruz Biotechnology, 1:5000), calbindin (polyclonal rabbit IgG CB-38a, Sigma, 1:10,000), or Ki67 (polyclonal rabbit NCL-Ki67p, Novocastra, 1:7000) antibodies at 4°C over night. Incubation in secondary antibody (rabbit anti goat 1:300, or goat anti rabbit 1:300, Vectastain) was followed by incubation with ABC solution (Vectastain). Finally 3,3′-diaminobenzidine (DAB) stained sections were mounted, DCX stained sections were counterstained with hematoxylin solution, and all sections were dehydrated and cover-slipped.

For the estimation of GC and apoptotic cell numbers, left hemispheres were embedded in 2-HEM (Technovit 7100, Kulzer GmbH). Hemispheres were washed with PBS and dehydrated in graded ethanols. After incubation in 1:1 ethanol and HEM solution, hemispheres were infiltrated in three consecutive HEM solutions for several days until final embedding. Twenty μm horizontal sections were cut and Giemsa-stained (Merck, Darmstadt, Germany) in 67 mmol KH_2_PO_4_ solution for 40 min at room temperature, differentiated in KH_2_PO_4_ for 90 s, dehydrated and cover-slipped. Mossy fiber terminals were Timm-stained following the procedure described before (Gatome et al., 2010).

### Lens weight and bone structure measurements for age determination

**Figure 2:**
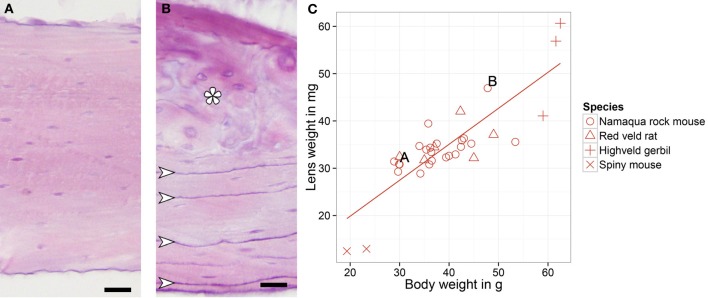
**Hematoxylin-eosin stained femur cross sections of a younger (A) and older (B) Namaqua rock mouse.** Adhesion lines in the outer circumferential lamellae (arrows) and the formation of osteons (asterisk) in the older animals indicate maturation and re-organization of bone tissue while aging. **(C)** Lens weight and body weight for all southern African rodents, letters indicate the animals whose bone structures are shown at the left. Scale bar: 20 μm.

The eye lenses were dried for 5 days at 80°C and weighted (Barker et al., 2003). Femurs were washed and immersed in a rapid decalcifying solution (Baker, Histo Grade) for 24 h. Using a digital calliper, a 3 mm segment was taken from the mid-diaphysis of the femur and infiltrated with HEM as described above. Twenty μm sections were hematoxylin-eosin stained. In the cross-sectioned femurs, adhesion lines (Figure 2) were counted in the inner and outer lamellar bone tissue using bright field and fluorescent illumination.

### Genotyping

Given the cryptic nature of some of the southern African murid rodent species, murid samples were genetically identified. This was critical for the red veld rat (*Aethomys chrysophilus*), which is morphologically indistinguishable from the Tete veld rat (*Aethomys ineptus*) (Skinner and Chimimba, 2005). In addition, the highveld gerbil (*Tatera brantsii*) is morphological similar to the recently re-classified *Gerbilliscus leucogaster* (formerly *Tatera*) which have not previously been genotyped from the sampling site. Extracts from bone tissue of all specimens of the subfamily Murinae, along with voucher specimens of *A. ineptus*, *M. namaquensis* (formerly *Aethomys*), and the re-classified *G. leucogaster* (subfamily Gerbillinae), were analysed molecularly by means of D-Loop amplification and sequence analyses. Primers L14925 and H16499 were used to target a 460 bp region of the mitochondrial genome, amplifying a segment of the D-loop gene (Bastos et al., 2011). Genomic amplification was performed in a 50 μl reaction volume while the thermal profile followed an initial denaturation step at 96°C for 20 s, 2 cycles of denaturation at 96°C for 12 s, annealing at 49°C for 30 s, and extension at 72°C for 2 min, 3 cycles of denaturation at 96°C for 12 s, annealing at 48°C for 30 s, and extension at 72°C for 2 min and 35 cycles of denaturation at 96°C for 12 s, annealing at 47°C for 30 s, and extension at 72°C for 2 min. All samples were purified using PCR Product Purification Kit (Roche), and cycle sequenced using BigDye v. 3.1 terminator cycle-sequencing kit (Perkim-Elmer, Foster City, USA). Samples were run on an ABI 3130 sequencer and the resulting sequence chromatograms were viewed and edited in Chromas program embedded in Mega 4 (Tamura et al., 2007). A BLAST nucleotide search (www.ncbi.nm.nih.gov/blast) was performed to identify the rodent reference species with the highest sequence identity. Additionally, phylogenetic analyses were inferred in Mega 4, in order to molecularly identify specimens according to their relationship with the voucher specimens. Of the 33 sampled animals, genotyping identified 22 as Namaqua rock mice, six as red veld rats, and three as highveld gerbils. The two spiny mice of the subfamily Deomyinae were easily morphologically identified without genotyping due to the presence of dorsal spines (Table 1).

### Quantification

The estimation of cell numbers followed the rules of design-based stereology. The optical fractionator (West et al., 1991) was applied using StereoInvestigator software (MicroBrightField, Inc., Williston, USA). Cells were counted using a 100× oil immersion lens (NA = 1.3). For DCX, on every 6th section (spiny mice every 5th section) the counting frame was set as 30 × 30 μm and step size as 150 × 150 μm. For GCs, on every 6th section the counting frame was set as 10 × 10 μm and step size as 210 × 210 μm.

Ki67-positive and pyknotic cells were counted exhaustively (asf = 1, tsf = 1) using a 100× oil immersion lens (NA = 1.3). Only cells within the GC layer and the subgranular cell layer were considered. Positive cells in the top focal plane were not counted. Every 6th section (spiny mice every 5th section) was analysed. GC size was measured using the Nucleator (MigroBrightField) with four test lines as described previously (Amrein and Slomianka, 2010).

### Statistical analyses

#### Southern African samples

Experimentally introduced variance in the cell number estimates was evaluated by calculating the Gundersen-Jensen Coefficient of Error (CE) using the conservative, *m* = 0 approach (Gundersen et al., 1999; Slomianka and West, 2005). The quality of the measurements was judged by the contribution of estimation procedure to group variances expressed by the ratio CE^2^/CV^2^ [CV = Coefficient of variation = Standard deviation (*SD*)/Mean cell count].

Statistical analyses of AHN were performed in SPSS Statistics version 20 software (IBM) using multivariate General Linear Models (GLM) with cell counts as a response variable and estimated age as co-variant. To compare AHN between the different species, the total number of Ki67- and DCX-positive cells was presented as a percentage of total GC numbers. Within Namaqua rock mice, the effect of pregnancy on AHN was tested using total cell counts as a response variable and estimated age as co-variant. In addition, relationship between age and neurogenesis-related cell counts and total GC number was tested using two-tailed Pearson *r* correlation analysis.

#### Habitat comparisons

For comparisons between warm and cold environments, we included data of the following European rodent species (all of the family Muridae; subfamily Murinae) from previous studies in our laboratory: Yellow-necked wood mouse (*Apodemus flavicollis*, *n* = 8) and pygmy field mouse [*Apodemus microps* (*uralensis*), *n* = 4] (Amrein et al., 2004a,b), both from the Tvier Rayon, Russia, long-tailed wood mouse (*Apodemus sylvaticus*, *n* = 9) (Hauser et al., 2009) and F1 of wild-trapped house mouse (*Mus musculus domesticus*, *n* = 7) (Klaus et al., 2012), both obtained from Zurich, Switzerland.

The four Apodemus species differ in their sociality and space use. Pygmy field mice are described as the least aggressive member of the Apodemus family (Suchomelova and Frynta, 2000) with extensive allogrooming (Stopka and Graciasova, 2001). Long-tailed wood mice form family groups with several females and one male, whereas the biggest Apodemus species, yellow-necked wood mice behave rather aggressive toward conspecifics (Niethammer and Krapp, 1978). The smallest mice, the pygmy field mice, inhabit open grass land. Long-tailed wood mice occupy bushes and hedges and the largest member of the family, the yellow-necked wood mice, are found close to or within forests (Niethammer and Krapp, 1978). The social structure in house mice is similar to long-tailed wood mice, but can vary between wild and commensal populations (Berry and Bronson, 1992).

All data were collected using the same markers, protocols, and analysis tools described for the southern African rodent species. Habitat classification follows the Köpper–Geiger climate classification (Peel et al., 2007). The southern African rodents live in a habitat described as temperate, with dry winter and hot summer (Cwa). The habitat of the rodents from Zurich and western Russia used in this study is described as cold, with warm summers and no dry seasons (Dfb).

Initially, General Linear Mixed Models (GLMM) were performed in JMP (SAS Inc.) with either ln(DCX/GC) or ln(Ki67/GC) as response variables and the fixed effects habitat, estimated age and the interaction of habitat with age. Individuals, nested within species, were included as a random factor. As this random factor explained less than 1% of the variance in AHN, it was not used for further analysis. In a second model, the fixed effect subfamilies (Deomyinae, Murinae, and Gerbillinae), estimated age and the interaction of subfamilies with age was included to test the effect of phylogenetic relatedness on AHN. Omission of the young European rodents with high AHN scores did not affect the level of significance of the results. All graphical presentations were made with *R* package ggplots2 (Wickham, 2010).

## Results

### Brain weight, body weight, and granule cell number

Comparing brain weight [*F*_(3, 29)_ = 27.57] and body weight [*F*_(3, 29)_ = 20.84] between species, Namaqua rock mice and red veld rats do not differ from each other (*P*_brainweight_ = 0.91; *P*_bodyweight_ = 0.47), otherwise, weights differ between all species (*P*_brainweight_ < 0.001; *P*_bodyweight_ < 0.001) (Table 1). Within Namaqua rock mice, there is no difference in these measurements between sexes [*F*_(1, 20)_ = 2.65; *P*_brainweight_ = 0.12; *F*_(1, 20)_ = 0.62; *P*_bodyweight_ = 0.44]. Due to a small sample size, gender differences in the other species were not evaluated statistically. In the hippocampal formation, the number of GCs [*F*_(3, 27)_ = 19.92] does not differ between Namaqua rock mice and red veld rats (*P* = 0.55), between Namaqua rock mice and highveld gerbils (*P* = 0.30), and between red veld rats and highveld gerbils (*P* = 0.20). Similarly, within Namaqua rock mice, there is no gender difference for total GCs [*F*_(1, 18)_ = 0.04; *P* = 0.84].

Spiny mice have exceptionally high numbers of GCs which differ from all other species (*P* < 0.001). This unexpected high number of GCs was verified by an independent second investigator, yielding results within 10% of the original estimate, i.e., within the margin of error defined by the CE estimates. To analyse this difference further, we estimated GC size. The cell diameter of spiny mice GCs is on average 6.8 μm, whereas GCs in Namaqua rock mice, red veld rats, and highveld gerbils measure on average between 7.9 and 8.2 μm in diameter.

### Age determination in wild small rodents

Aging wild-trapped small mammals is not trivial, and, without a calibration curve, as used by Epp et al. (2009), it can only be relative. We measured lens weight and total bone lines for each individual animal (Figure 2) as suggested by Barker et al. (2003) and Epp et al. (2009). The data were recalculated as a percentage of the mean of the respective measurements within species and summarized in an averaged numerical rank. The transition into an estimated age in months is based on the dataset of Namaqua rock mice. Assuming that all animals have wintered, we gave the animal with the lowest rank the estimated age of 4 months. Twenty-one out of twenty-two animals showed a continuous rank increase, indicating that these animals were born during the preceding season; the oldest animal of this continuous rank line is therefore set to be 12-months old. A linear function was calculated and applied to transform ranks into estimated age in months in all animals. By so doing, 31 animals had an estimated age of between 4 and 12 months, one red veld rat and one Namaqua rock mouse were likely to be older than 1 year.

### Proliferation and differentiation in southern murid African rodent: effect of species and age

**Figure 3:**
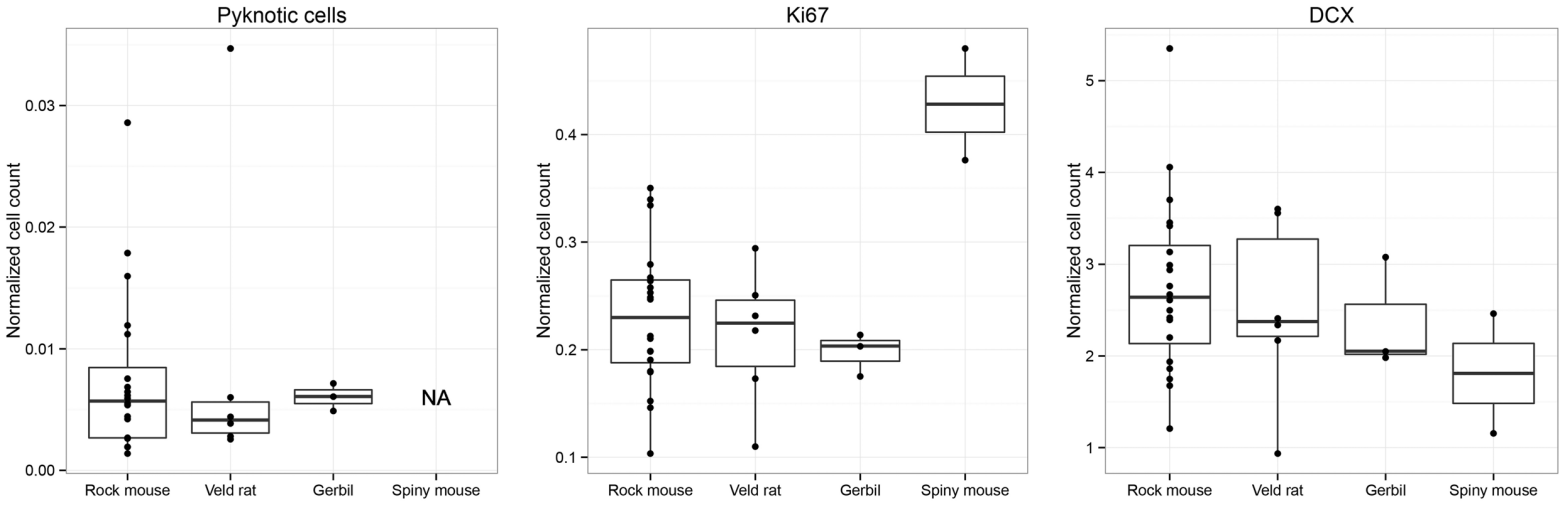
**Boxplots of the distribution of estimated numbers of pyknotic cells, proliferating cells (Ki67), and young cells of the neuronal lineage (DCX) relative to total granule cells, i.e., normalized cell numbers.** Southern African species do not differ from each other in any measurement, except for spiny mice which have exceptionally high ratios of proliferating cells in the dentate gyrus.

Despite the morphological differences summarized above, the total number of proliferating cells (Ki67; Table 1, Figure 3), once normalized to the resident GCs [*F*_(3, 27)_ = 7.91], do not differ significantly between Namaqua rock mice and red veld rats (*P* = 0.55), between highveld gerbils and Namaqua rock mice (*P* = 0.40), and between highveld gerbils and red veld rats (*P* = 0.73). Only spiny mice show a higher percentage of proliferating cells than all other species (*P* < 0.001). The percentages of differentiating cells of neuronal lineage and dying cells within the dentate gyrus do not differ between species [DCX: *F*_(3, 27)_ = 0.71; *P* values between 0.83 and 0.15; dying cells: *F*_(2, 26)_ = 0.16; *P* values between 0.76 and 0.59; the number of dying cells in spiny mice was not estimated].

Across all species, there was no correlation between estimated age and normalized proliferating cells [*r*_(29)_ = −0.18, *P* = 0.33], dying cells [*r*_(27)_ = −0.30, *P* = 0.11], or total GCs [*r*_(29)_ = 0.11, *P* = 0.56], whereas normalized numbers of DCX-positive cells show a negative correlation with age [*r*_(29)_ = −0.41, *P* = 0.02]. Within the largest animal sample (*n* = 22), the Namaqua rock mice, we tested cell counts without normalization and found a weak negative correlation between age and DCX-positive cells [*r*_(20)_ = −0.41, *P* = 0.06], and there were no correlations for proliferating cells [*r*_(20)_ = −0.30, *P* = 0.17], apoptotic cells [*r*_(18)_ = −0.23, *P* = 0.32], or total GCs [*r*_(18)_ = −0.21, *P* = 0.38] with age.

### Effects of previous pregnancies in Namaqua rock mice

**Figure 4:**
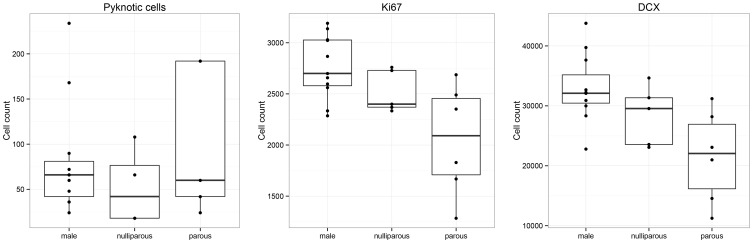
**Within Namaqua rock mice, females that previously had one or several litters (parous) show reduced number of proliferating cells (Ki67) and young neuron (DCX) compared to males.** Cell number estimates of parous females are statistically not different from nulliparous females.

Female Namaqua rock mice with previous pregnancies (parous) harbor less proliferating cells than males [*F*_(2, 19)_ = 5.78; *P* = 0.004], and similar numbers as nulliparous females (*P* = 0.14). Similarly, the number of DCX-positive cells in parous females was reduced compared with males [*F*_(2, 19)_ = 5.37; *P* = 0.006], but not different from nulliparous females (*P* = 0.30) (Figure 4). Nulliparous females did not differ from males for either proliferating cells (*P* = 0.19) or DCX-positive cells (*P* = 0.11). For the number of dying cells [*F*_(2, 19)_ = 1.66], the only trend is found in parous females compared to nulliparous females (*P* = 0.09). The number of total GCs did not differ between males, nulliparous, and parous females.

### Effect of habitat and phylogeny

**Figure 5:**
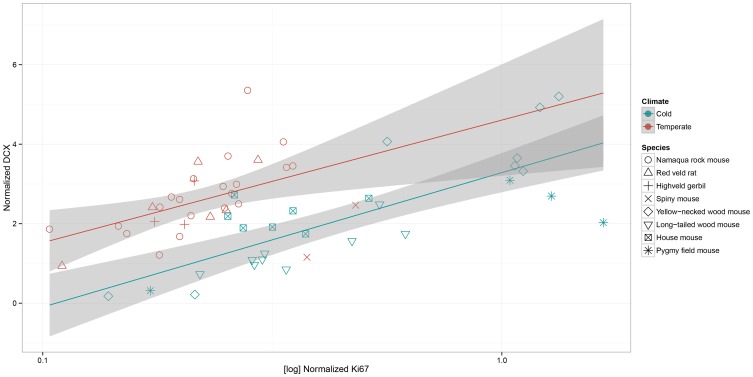
**In the scatterplot of normalized numbers of young differentiating neurons (estimated number of DCX-positive cells relative to the total granule cell number) to log-transferred normalized proliferating cell (estimated number of Ki67-positive cells relative to the total granule cell number) the species from the cold European climate (Dfb) are separated from those of the general warm southern African habitat (Cwa), which is described as temperate in the Köpper–Geiger climate classification.** Southern African species show lower proliferation (*P* = 0.0003), but higher neuronal differentiation (*P* = 0.0036) than the rodents from the cold climate. Shaded areas indicate the 95% confidence interval.

The four southern African rodent species exhibited a different pattern of AHN compared to the four rodent species from Europe (Figure 5). In rodents from the southern African temperate climate, characterized by dry winter and hot summer (Köpper–Geiger climate classification, Cwa), normalized proliferation is decreased [*c*^2^(1, *N* = 59) = 13.24, *P* = 0.0003]. Normalized number of DCX-positive cells is increased [*c*^2^(1, *N* = 59) = 8.47, *P* = 0.0036] compared to rodents from the European cold climate (Köpper–Geiger climate classification Dfb). There is no habitat by age interaction for proliferation [*c*^2^(1, *N* = 59) = 2.57, *P* = 0.1087] and DCX-positive cells [*c*^2^(1, *N* = 59) = 1.34, *P* = 0.2469].

Subfamily membership (Murinae: including all species from Europe and Namaqua rock mice and red veld rat from southern Africa; Deomyinae: the spiny mice; and Gerbillinae: highveld gerbils) did not yield significant effects [DCX: *c*^2^(2, *N* = 59) = 0.22, *P* = 0.896; Ki67: *c*^2^(2, *N* = 59) = 1.06, *P* = 0.588].

### Precision of stereological estimates

The precision of the stereological estimates was calculated for the data on Namaqua rock mice, red veld rats, and highveld gerbils. The CE of total GC numbers, proliferating cells (Ki67), and cells of neuronal lineage (DCX) ranged between 0.06 and 0.1, indicating that the estimation procedures generated a variance that amounts to only 6–10% of the mean. Due to the low numbers of dying cells, CEs for these estimates ranged between 0.3 and 0.36. For all estimates, the methodologically introduced variance is a minor contributor to the total variance of the group means (CE^2^/CV^2^ < 0.14).

### Qualitative hippocampal characteristics in Namaqua rock mice and red veld rats

**Figure 6:**
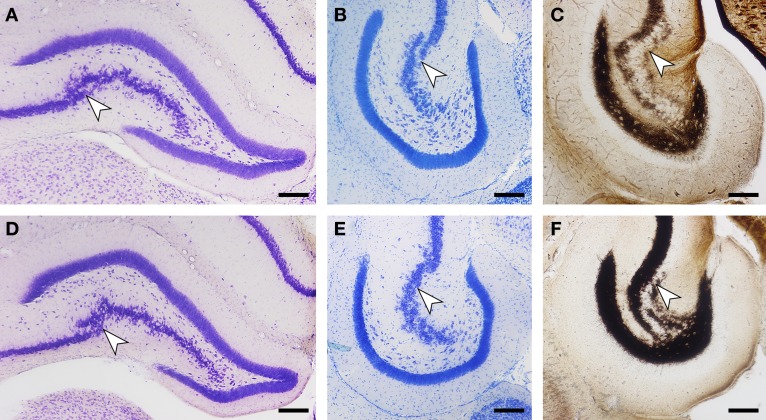
**Histological stains of the hippocampus in Namaqua rock mouse (A–C) and red veld rat (D–F) reveal common, for murine rodents unusual features.** In coronal **(A,D)** and horizontal **(B,E)** sections of Giemsa-stained material, a transition from CA3 to CA4 pyramidal cell layer (arrow) can be distinguished. Timm staining **(C,F)** shows that the infra- and intra-pyramidal terminal field is largely restricted to the CA4 region. Scale bar: 200 μm.

**Figure 7:**
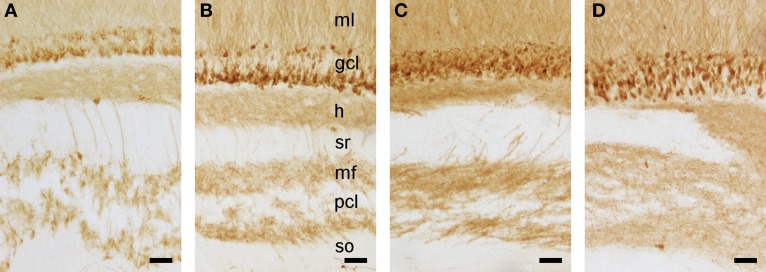
**Calbindin stained detail of the hippocampus in Namaqua rock mouse (A), red veld rat (B), highveld gerbil (C), and spiny mouse (D).** While mature granule cell and their processes express this protein in all four species, expression is, in contrast to that in laboratory mice and rats, very heterogeneous: tiered in Namaqua rock mouse and red veld rat, mosaic in spiny mouse, and intermediate between these two forms in the highveld gerbil. Molecular layer (ml), granule cell layer (gcl), hilus (h), stratum radiatium (sr), mossy fibers (mf), pyramidal cell layer (pcl), stratum oriens (so). Scale bar: 50 μm.

As described above, we did not observe a quantitative difference in brain weight, body weight, or any of the assessed cell numbers between red veld rats and Namaqua rock mice. Histologically, both species show a reflected blade (CA4) of the CA3 pyramidal cell layer as it inserts into the dentate hilus and joins the infrapyramidal limb of the polymorphic cell layer of the hilus (Figure 6). Timm-stained intra- and infra-pyramidal mossy fiber buttons are largely restricted to CA4-like pyramids. Both Namaqua rock mice and red veld rats were also characterized by a distinct differential distribution of calbindin within CA1 pyramidal cells along the proximo-distal axis of the cell layer, and an increase in the apparent number and staining intensity of calbindin-positive cells toward the temporal hippocampus (not illustrated). Interestingly, a heterogenous distribution of calbindin in GCs (Figure 7) were observed in Namaqua rock mice, red veld rats (both stratified), and spiny mice (mosaic).

## Discussion

AHN in laboratory rodents is affected by a multitude of factors, and it is surprisingly easy to increase or decrease the number of dividing or differentiating cells in laboratory rodents. This plasticity has led to concerns about the physiological importance of AHN, as key physiological parameters tend to be kept within a narrow range (Lazic, 2012). Compared to laboratory animals, the plasticity of AHN in wild rodents is much smaller—both for baseline levels in outbred species groups (Klaus and Amrein, 2012) and in response to experimental challenges (Hauser et al., 2009; Klaus et al., 2012; Schaefers, 2013). These observations not only mitigate the concern raised above, they also raise the possibility that the extent of AHN can be related to aspects of the physiology, ecology, or behavior that characterize a species rather than to acute events in the life of its members. A cross-correlational comparative approach that may elucidate the adaptive functional significance of AHN does require one to follow the suggestion of Nottebohm (2002) to “look more closely at adult neurogenesis … in a diversity of free-ranging animals leading a normal life.” Toward this aim, we here quantitatively define proliferation and neuronal differentiation at the stage of DCX expression in several southern African rodent species that share the same habitat, but differ from each other both in phylogeny and ecology.

The southern African rodents in this study were sampled from the same locality, which is characterized by strong seasonality. As an adaptation, all four rodent species breed seasonally and have litters only in the wet, warm summer months (Fleming and Nicolson, 2004; Skinner and Chimimba, 2005; Muteka et al., 2006). Apart from these common features, the species differ in their feeding patterns, territory use, and social structure. The Namaqua rock mouse has the widest geographical distribution throughout the southern African subregion with a habitat preference to rocky crevices, outcrops, and rock strewn areas (Skinner and Chimimba, 2005). The Namaqua rock mouse is omnivorous, but preferably lives on seeds, grass, and other vegetation (Mills and Hes, 1997; Kingdon, 2001). This species is polygynous, but may also live in small colonies (Fleming and Nicolson, 2004; Muteka et al., 2006). The Namaqua rock mouse shows no sexual dimorphism, but male territories, overlapping with female territories, are up to 50% larger than those of females (Fleming and Nicolson, 2004; Skinner and Chimimba, 2005).

The red veld rat has a narrower geographical range, but commonly coexists with the Namaqua rock mouse (Linzey and Chimimba, 2008). In contrast to the Namaqua rock mouse, red veld rats are not gregarious, and live either in pairs or family parties (Skinner and Chimimba, 2005). The red veld rat differs from the Namaqua rock mouse in the preferred microhabitat (Linzey and Chimimba, 2008).

The highveld gerbil studied here tolerates more arid conditions than other gerbils. Colonies of highveld gerbils live in an extensive burrow system, which can extend up to 70 ha, and, similar to Namaqua rock mice, males tend to have larger territories than females (Moor, 1969; Skinner and Chimimba, 2005). In contrast to both the Namaqua rock mouse and the red veld rat, the highveld gerbil is omnivorous, feeding on seeds, plants, and insects.

The spiny mouse is the only precocial rodent investigated here. It shares with the highveld gerbil the omnivorous feeding pattern. Spiny mice live solitary or in family parties (Skinner and Chimimba, 2005).

Despite these ecological differences, the four southern African rodents, representatives of three different subfamilies, harbor a similar percentage (~2%) of DCX-positive cells in their GC layer. In view of the differences in both phylogeny and ecology of the species, the stability of this proxy for AHN and the differences between the African and European groups point toward habitat as an important factor in the shaping of proliferation and differentiation as part of AHN beyond species-specific adaptations and across murid taxonomic units.

### Closely related species: Namaqua rock mouse and red veld rat

Namaqua rock mice and the red veld rat are considered phylogenetically closely related (Mills and Hes, 1997; Skinner and Chimimba, 2005). None of the AHN-associated cell estimates differ between the two species. No obvious qualitative morphological differences were observed in the hippocampal histoarchitecture, the distribution of mossy fibers or calbindin. The hippocampus shows a reflected blade of the CA3 cell layer into the hilar region [CA4 after Lorente de Nó's terminology (Lorente de No, 1934)], a feature present in, for example, primates (Rosene and Van Hoesen, 1987), rabbits and guinea pigs (Geneser-Jensen et al., 1974; Geneser, 1987), or foxes (Amrein and Slomianka, 2010). A CA4-like cell layer has not previously been observed in murine rodents. This CA4-like cell layer is accompanied by suprapyramidal and infrapyramidal mossy fiber projections that are typical for the most proximal CA3 pyramidal neurons in other murine rodents (Schwegler and Lipp, 1983). Further investigation is necessary to determine if this field in Namaqua rock mice and red veld rats shares structural and connective characteristics that define CA4 in other species.

### Aging in small wild rodents and its correlation with AHN

All animals were sampled after winter and before the seasonal breeding starts (Withers, 1983; Muteka et al., 2006), and it is therefore highly unlikely that any animal would be younger than 4 months. By comparing the range of lens weights in Namaqua rock mice, we observe a 63% increase in lens weight between the presumably youngest to the oldest animal. As this value reflects a single animal that most likely overwintered twice, we tested for lens weight differences in the animals that overwintered once. We observe a 37% increase in lens weight between the presumably youngest to the oldest animals in this cohort, which would correspond to an age difference of more than 4 months in adult Sprague-Dawley rats (Epp et al., 2009). We therefore expect to have considerable age variation in our sample of Namaqua rock mice. Testing for age-dependent regulation of AHN, we found that the number of DCX-positive cells in the southern African rodents declines with age, as it has previously been reported for many mammals (Kuhn et al., 1996; Amrein et al., 2011). However, the number of proliferating cells showed no correlation with age. It is not clear if this correlation has been obscured by variance associated with small cell numbers and a tentative age ranking. Alternatively, proliferation in these species may be low, but stable in animals born in the same season as it has previously been shown in wood mice (Amrein et al., 2004b).

### Lasting effect of pregnancy in Namaqua rock mice

Within Namaqua rock mice, we observed that females that had previously experienced at least one pregnancy (parous) show lower AHN than males. The small sample of nulliparous females do not differ from either males or parous females. Our observations are in general agreement with previous studies, in which reproductively active female meadow voles showed lower proliferation compared to both males and reproductively inactive females (Galea and McEwen, 1999; Ormerod and Galea, 2001). Studies in laboratory rodents reported a decrease in neurogenesis during gestation and the post-partum period, with a recovery of AHN to baseline levels after weaning (Leuner et al., 2007; Pawluski and Galea, 2007; Kim et al., 2010). Our animals were sampled at the beginning of the breeding season, and the last post-partum period must have passed ~3 months before. A persisting decrease in AHN associated with a life history event has previously been shown in shrews, in which AHN completely ceases after overwintering (Bartkowska et al., 2008).

### The effect of habitat

Behavioral flexibility which would allow quick adaptations to environmental challenges might benefit from AHN (Amrein and Lipp, 2009; Garthe et al., 2009; Amrein et al., 2011). If the types and frequencies of such challenges are rather stable in a habitat, one may expect that there is little need for an extensive modulation of the extent of AHN. AHN in wild or wild-derived wood mice and house mice is indeed relatively stable (Hauser et al., 2009; Klaus et al., 2012; Schaefers, 2013). In the sympatric southern African rodents presented here, the stability in proliferation and neuronal differentiation is maintained across three murid taxonomic units. By comparing the southern African rodents with mice from a cold climate, we show here that rodents from different habitats show distinct patterns. Southern African rodents generate less new cells, but in proportion to proliferation, harbor more DCX-positive cells than rodents from Europe. The European species presented here are monophyletic (i.e., all species belong to the subfamily Murinae). It cannot be ruled out that phylogenetic inertia is partially responsible for similarities between these species (Harvey and Pagel, 1991; Blomberg and Garland, 2002). The southern African Murinae (Namaqua rock mouse and red veld rat) do however not cluster with the European ones, and phylogenetic inertia can therefore only be responsible for similarities in one of the two murine species groups. The habitat-dependent differences are also found in the species pair Namaqua rock mouse and long-tailed wood mouse, even though they are considered to be ecological equivalents in their respective habitats. Low proliferation rates, but high numbers of DCX-positive cells have previously been observed in other species (Amrein and Slomianka, 2010). At the cellular level, this can be achieved by either increasing survival rate of the cells, extending the maturation phase of young neurons, or a combination thereof. In our study, apoptotic cells occur less frequently in the African rodents than in the European species, but the variance in the apoptosis data is too high to draw firm conclusions on the survival of newly born cells. Species-specific differences in maturation time have previously been reported. Young neurons mature slower in mice than in rats (Snyder et al., 2009), and the maturation of newly born hippocampal neurons in primates may take more than 6 months (Ngwenya et al., 2006; Kohler et al., 2011). Independent of the regulatory mechanisms, southern African rodents in this study harbor more DCX-positive cells in the hippocampus than rodents from Europe. Behavioral tests in laboratory mice indicate that AHN is not necessary for learning *per se* (Jaholkowski et al., 2009) but required for fast behavioral changes that are interpreted as increased cognitive flexibility (Garthe et al., 2009; Kempermann, 2012). We previously suggested that neuronal differentiation can mediate species-specific adaptations to habitat requirements (Amrein et al., 2011). The obvious question is therefore which habitat factor drives the development of the observed differences? Based on the Köpper–Geiger habitat classification, which includes temperature and rainfall, the seasonally high temperatures and water shortages in the southern African habitat clearly differ from the colder European habitat with no shortage in water. Environmental challenges for small mammals in the hot, dry conditions of deserts exceed the ones for animals living in cold climates, e.g., the upper critical temperature for an euthermal mammal is less variable than the lower critical temperature, and also water conservation requires extensive adaptations on the physiological and behavioral level (Merritt, 2010). If and how these factors might interact with AHN is unclear. Many parameters other than temperature and metabolic water homeostasis that are linked or independent from climatic conditions, such as food availability or predator risk, could play a role in regulating AHN. We provide evidence that the habitat can shape two of the stages that comprise AHN, which implies that the specific pattern expressed provides a selective advantage. Further studies are needed to define the interactions between habitat and hippocampal function that provide this advantage.

### Conflict of interest statement

The authors declare that the research was conducted in the absence of any commercial or financial relationships that could be construed as a potential conflict of interest.
